# Anthony Staines: public health leader, scholar and advocate for a just society

**DOI:** 10.1093/eurpub/ckag171

**Published:** 2026-10-29

**Authors:** Tit Albreht, Charlotte Marchandise, Martin McKee

**Affiliations:** President of EUPHA; Executive Director of EUPHA; The London School of Hygiene & Tropical Medicine (LSHTM)

The European public health community mourns the death of Anthony Staines, Professor of Health Systems at Dublin City University, who died on 27 August 2026. Anthony was an epidemiologist, health-systems scholar and social activist. Yet the first thing colleagues remember is simpler: he was one of the good ones. Tributes shared on EUPHA networks have begun with his kindness, gentle manner, generosity and dedication. These qualities were central to how he worked and to his influence across Ireland and Europe.

Anthony’s European contribution was most visible in his leadership of two European Public Health Conferences. He led the 14th conference in 2021, originally planned for Dublin but moved online because of COVID-19. The circumstances could scarcely have been more difficult. Anthony nevertheless helped turn adversity into an inclusive European meeting, sustaining professional connection, the exchange of evidence and a sense of solidarity when all three were urgently needed.

Two years later, he chaired the 16th Conference, held in person in Dublin in November 2023. Its theme, ‘Our Food, Our Health, Our Earth: A Sustainable Future for Humanity’, reflected his ability to connect immediate health concerns with the structural challenges facing society. In parallel, he led the Dublin statement on food, environment and planetary health [1]. Together, the two meetings demonstrated his adaptability, persistence and profound commitment to Europe’s public health community.

That commitment continued away from the conference platform. When the European Public Health Conference faced a severe financial crisis after the loss of an EU operating grant, Anthony stepped forward. He joined weekly meetings over many months, always having read the forecasts and papers and always searching for new ideas. Just as importantly, he cared for the team working on the recovery. And he never lost sight of the purpose of the enterprise: the communities public health exists to serve, especially those who are underserved and too often unheard.

The many tributes that have poured in to EUPHA describe a ‘public health legend’, a scientist and humanitarian, a true gentleman and a wise, inspiring figure who found the right words at the right time. Former students recalled his exciting teaching, exceptional care and passionate mentorship. Others remembered his almost magical ability to connect with people and help whoever needed him. As one colleague observed on a rainy Dublin morning, Irish tradition would have Anthony happy and smiling down on them all.

Anthony graduated in medicine from Trinity College Dublin in 1984 and initially trained in paediatrics. He later moved into public health and epidemiology, completing an MSc at the London School of Hygiene & Tropical Medicine and a PhD in spatial epidemiology at the University of Leeds. After working in Leeds and at Imperial College London, he returned to Ireland in 1997 to join University College Dublin. In 2007, he became Dublin City University’s first Chair of Health Systems.

His research encompassed child health, disability, cancer epidemiology, health services, integrated care and digital transformation. A recurring concern was using health information to create fairer, safer and more responsive services. He worked closely with Ireland’s Health Service Executive, including through a secondment to develop an information technology strategy for the health service.

During the pandemic, Anthony brought epidemiological evidence directly into public debate. In July 2020, he co-founded Ireland’s Independent Scientific Advocacy Group [2]. He argued for effective measures, clear communication and solidarity with those at greatest risk. He later warned against complacency and called for stronger public-health institutions and better preparedness [3].

Anthony’s public service extended far beyond academia. He chaired the Irish Blood Transfusion Service and St Michael’s House, a major provider of services for people with intellectual disabilities. He served on Ireland’s Higher Education Authority and Health Insurance Authority and contributed to organizations supporting people affected by cancer and by drug- and alcohol-related harms. He also supported the campaign for marriage equality and engaged openly in political life.

He was deeply committed to Ireland and to making it a more inclusive country. He described himself simply as ‘a doctor, an academic, and a social activist’. His guiding conviction was equally direct: ‘I believe that social solidarity matters, and that we need a more equal, and more just society’. He was horrified by the damage done by Brexit to relations on the island of Ireland [4].

Anthony is survived by his wife Úna, his children, Lorna and Jonathan, and his wider family. EUPHA extends its deepest sympathy to them, to his colleagues and students, and to his many friends in Ireland and across Europe. We remember a scholar who made evidence useful, a leader who never forgot the people behind institutions, and a generous colleague whose kindness touched countless lives. His contribution will endure in the people he mentored, the institutions he strengthened, and the more just society he worked to create.
